# Managing head and neck cancer patients during the COVID-19 pandemic: the experience of a tertiary referral center in southern Italy

**DOI:** 10.1186/s13027-021-00352-9

**Published:** 2021-02-05

**Authors:** Francesco Longo, Eleonora M. C. Trecca, Aurelio D’Ecclesia, Chiara Copelli, Karim Tewfik, Alfonso Manfuso, Nicola Pederneschi, Annalisa Mastromatteo, Matteo Aldo Russo, Antonio Pansini, Luca M. Lacerenza, Pier Gerardo Marano, Lazzaro Cassano

**Affiliations:** 1 Department of Maxillofacial Surgery and Otolaryngology, IRCCS Casa Sollievo della Sofferenza, Viale Cappuccini, 1, 71013 San Giovanni Rotondo (Foggia), Italy; 2 Department of Otolaryngology- Head and Neck Surgery, University Hospital of Foggia, Foggia, Italy; 3 Department of Surgical Sciences; Unit of Maxillofacial Surgery, University Hospital of Turin- Città della salute e delle scienze, Turin, Italy; 4 Department of Neurosciences, Reproductive and Odontostomatological Sciences, Maxillofacial Surgery Unit Naples, University of Naples Federico II, Naples, Italy

**Keywords:** Coronavirus disease, Critical care, Hospital administration, Ambulatory care, Primary health care, Otolaryngology, Maxillofacial surgery, Head and neck cancer, Infection

## Abstract

**Background:**

The medical community has been deeply involved in fighting the Coronavirus disease 2019 (COVID-19) pandemic and, as a consequence, the care of non-COVID-19 patients has been impacted. However, the treatment of head and neck cancer patients is not deferrable, and an integrated strategy is required. The aim of the current article is to present the experience in the management of head and neck patients during the COVID-19 pandemic at the research hospital “Casa Sollievo della Sofferenza”. This review contains replicable and widely usable instructions on how to avoid delays in the diagnosis and treatment of head and neck tumors and to ensure a gradual return to elective procedures.

**Main text:**

The Head and Neck Department of the research hospital “Casa Sollievo della Sofferenza” includes an Otolaryngology and a Maxillofacial Surgery Unit, both of which deal with the diagnosis and treatment of benign and malignant pathologies of the head and neck, as well as urgent/emergent consultations and surgical procedures that necessitate time sensitive operative management, such as cochlear implantation (CI). Given these premises and the complexity of the Department, the “COVID-19 organizing protocol” of the research hospital “Casa Sollievo della Sofferenza” was divided into two phases in accordance with the different stages of the pandemic and the priority of treatment. Special attention was given to the medical surveillance of health care workers and hospitalized patients, to the organization of the outpatient clinic and the operating setting as well as to the implementation of telehealth systems.

**Conclusions:**

The COVID-19 pandemic is going to be a long-term situation with lasting effects on the public health and the entire society. Therefore, an efficient health care system has to adopt a double strategy: always being ready for a “new wave” of the pandemic and not forgetting non-COVID-19 patients, among whom head and neck cancer patients represent a priority. More than 1 year since the first outbreak in Wuhan, this review offers a unique and helpful perspective that incorporates awareness of the disease.

## Introduction

Coronavirus Disease 2019 (COVID-19) is a global pandemic caused by the novel severe acute respiratory syndrome coronavirus 2 (SARS-CoV-2) infection, which is responsible for a wide range of mild to severe symptoms of the respiratory tract and multiple organ systems. In its most severe forms, the treatment of COVID-19 involves a multi-specialist approach and an integrated strategy [1]. Since the first cases in December 2019 in Wuhan, China, the medical community has been deeply involved in fighting this unprecedented pandemic and, as a consequence, the management of non-COVID-19 patients has been profoundly impacted. A survey conducted by the Italian Society of Otolaryngology-Head and Neck Surgery (SIOeChCF) reported a decrease in the number of elective otolaryngology (ORL) activities by 81.24% and a conversion of the Italian ORL departments in COVID-19 units in 22% of cases [2], while another study evidenced a reduction in the number of ORL emergency consultations by 80.8% [3]. Although the treatment of many Ear, Nose and Throat (ENT) conditions could be deferrable without any consequences, this is not the case in head and neck cancer patients whose care has to be prioritized during these difficult times [4]. Besides maintaining high-quality standards of treatment, the other main priority of regional cancer hubs has been the prevention of COVID-19 nosocomial infections. In addition to guaranteeing patients’ safety, the protection of physicians and health professionals plays a role of primary importance in limiting the spread of the disease [5]. Otolaryngology-head and neck surgery (ORL-HNS) has been recognized as one of the specialties at highest risk since March 2020 [6] due to the symptoms of upper respiratory tract infections (URTI) which can be similar to the manifestations of COVID-19 (i.e. smell and taste dysfunction [7, 8], dysphonia [9]) and to the invasive nature of examinations (i.e. nasal endoscopy) and aerosol-generating procedures (AGPs) (i.e. functional endoscopic sinus surgery [10], tracheostomy [11], mastoidectomy [12], orthognathic surgery [13]). The international registry of Otolaryngologists-head and neck surgeons promoted by the Young Otolaryngologists–International Federation of Otolaryngologic Societies (YO-IFOS) has identified 361 Otolaryngologists reporting COVID-19 at the time of publication in May 2020 and, unfortunately, this number has increased worldwide during the last months [14]. Another multicentric survey conducted in 23 Italian maxillofacial surgery departments found that 4% of maxillofacial surgeons and 22% of maxillofacial surgery residents tested positive for COVID-19 [15].

Given these premises, we would like to present our experience in the management of head and neck patients during the COVID-19 pandemic at the research hospital “Casa Sollievo della Sofferenza”, which has been recognized as the best hospital of Southern Italy, according to the annual Newsweek report [16] and is located in one of the hardest hit provinces of the Puglia region. The main purpose of this review is to provide replicable and widely usable recommendations in order to avoid delays in the diagnosis and treatment of head and neck tumors, which can be life changing for the patients both in terms of mortality and quality of life, while ensuring a gradual return to elective procedures.

## The “COVID-19 organization protocol” at the research hospital “Casa Sollievo della Sofferenza”

The “COVID-19 organization protocol” at the research hospital “Casa Sollievo della Sofferenza” was created in cooperation with the Preventive Medicine and Hygiene Section of the Institution and in accordance with the guidelines from the Higher Institute of Health (ISS).

The Head and Neck Department includes an Otolaryngology and a Maxillofacial Surgery Unit, both of which deal with the diagnosis and treatment of benign and malignant pathologies of the head and neck, such as benign and malignant tumors of the salivary glands, the oral cavity, the skin, the larynx, the paranasal sinuses and anterior skull base. Free flap surgery is executed by the Maxillofacial Surgeons for the reconstruction of complex head and neck defects and facial reanimation. The anterolateral thigh and radial forearm free flaps are the most commonly performed procedures at our institution, 50 and 49 flaps on average per year, respectively according to a recent case review [17]. Additionally, the Head and Neck Department performs urgent/emergent consultations (i.e., maxillofacial traumas, vertigo, inflammatory diseases, foreign bodies, airway obstructions, sudden hearing loss, epistaxis, etc.), which requires a high priority level within the hospital administration. Besides the ORL emergencies and cancer patients, the Head and Neck Department is also a referral center for other surgical procedures, such as cochlear implantation (CI), that necessitate time sensitive operative management [18]. Given these premises and the complexity of the organization, an integrated strategy was requested in order to provide adequate treatment to every patient within an appropriate timeframe.

### Medical surveillance of health care workers and hospitalized patients during the COVID-19 pandemic

The safety of health care workers plays a central role in guaranteeing the best possible treatments to patients. The Head and Neck Department currently employs one Director, 10 otolaryngologists, 6 maxillofacial surgeons, 3 resident physicians, 22 registered nurses, 4 speech therapists, 3 audiometric technicians, 10 healthcare operators and one health coordinator. The hospital requires health care workers to undergo nasal/oropharyngeal swabs for the detection of COVID-19 every month, in case of suspicion/contact with positive individuals, and for those returning after travelling outside the Puglia region. Specifically, molecular tests are the first choice at our hospital for the diagnosis of SARS-CoV-2 infection and the procedure comprises the execution of a double nasal/oropharyngeal swab. The nasal/oropharyngeal swabs are routinely performed by the otolaryngologists on patients/healthcare workers, and otolaryngologists are subjected to swabs for diagnosis, as well. The support of the ENT team is fundamental for minimizing the false negative rate among COVID-19 positive individuals [19].

Patients admitted to the Head and Neck Department have to test negative for COVID-19 in order to be hospitalized or must undergo a swab test, if referred for emergency ORL consultations by the Emergency Department. Patient visitors are routinely not allowed in the Department; exemptions are granted upon request for valid reasons. In case of undeferrable ENT conditions, such as dyspnea, hemorrhage, which require immediate treatment, without the possibility to perform a swab test, patients are considered positive and temporarily hospitalized in a distinct “COVID-19 gray area”. This area has a dedicated patient room/operating room with separate entrances and negative pressures where suspected patients can receive the appropriate treatment and/or undergo the life-saving surgical procedure while waiting for the response of molecular test (usually 3–6 h). In such cases, the health care personnel wear personal protective equipment (PPE) indicated by the guidelines of the SIOeChCF for tracheostomies [20]/surgical activities [21] which consists of FFP2 or preferably FFP3 mask, face shield, double gown, double gloves, double cap and shoe cover. This was the recent case of a patient (male; 53 years old) affected by a laryngeal malignant tumor classified as IVA according to American Joint Committee on Cancer (AJCC) Cancer Staging Manual (8th Edition), who firstly underwent a tracheostomy in the “COVID-19 gray operating room” and was subsequently hospitalized in the Head and Neck Department after testing negative. Two days after the tracheostomy, he could safely undergo a total laryngectomy with bilateral cervical lymphadenectomy without any delay in diagnosis and treatment.

Also, it is worth noting that head and neck cancer patients warrant additional surveillance during the COVID-19 pandemic as they are particularly susceptible to pulmonary infections and respiratory complications [22]. This was the recent case of a patient (male; 50 years old) hospitalized in the Head and Neck Department who underwent an open partial horizontal laryngectomy (OPHL) type IIa and developed post-operative pneumonia documented by computed tomography (CT). After excluding bacterial and fungal etiologies, a high level of suspicion for SARS-CoV-2 infection was raised and the patient was isolated while undergoing several tests in cooperation with the Internal Medicine Department. Besides blood tests for the differential diagnosis (i.e., Cytomegalovirus, Epstein-Barr virus), the patient underwent two other swab tests and serology testing for COVID-19 after 1 week, all of which were negative. Bronchoalveolar lavage (BAL) was performed to definitively exclude COVID-19 and the patient was able to stop isolation without any risks to the other patients and health workers.

Three registered nurses and one Otolaryngologist tested positive for COVID-19 thus far, while no hospitalized patients with COVID-19 have been identified. Therefore, it is possible that the health care workers who tested positive contracted the disease outside the hospital or in a location other than the Head and Neck Department. Certainly, this vigilant medical surveillance limited the spread of the disease and the occurrence of an outbreak so that even during the Italian lockdown, interruption to the activities of the Head and Neck Department or its conversion in COVID-19 unit, was never required.

### Guide to provide care during the COVID-19 pandemic

The “COVID-19 organizing protocol” at the research hospital “Casa Sollievo della Sofferenza” was divided into two phases in accordance with the different stages of the pandemic and the priority of treatment:

- *Phase 1 protocol* (carried out from March 2020 to May 2020): The Head and Neck Department was active only for urgent/emergent surgical procedures and consultations, as well as for the management of undeferrable oncologic patients, including those scheduled for reconstructive procedures.
- *Phase 2 protocol* (from June 2020 to present): The Head and Neck department is gradually returning to the clinical and surgical routine of the pre-COVID-19 era. Besides the procedures and consultations included in the “phase 1 protocol”, the surgical procedures scheduled in this phase include at least one CI per week, as well as tympanomastoidectomy for chronic ear disease/cholesteatoma prioritizing cases with imminent complications, removal of benign tumors of head and neck, orthodontic-orthognathic surgery in disabled patients, functional endoscopic sinus surgery (FESS) including dacryocystorhinostomy (DCR) for inflammatory disease priority cases with impending complications, sleep surgery including transoral robotic surgery (TORS) priority patients affected by severe obstructive sleep apnea syndrome (OSAS) patients (apnea hypopnea index, AHI: > 30). Table 1 demonstrates the changes of head and neck pathologies presented by hospitalized patients at our department before and during the COVID-19 pandemic.

**Table 1:** Head and neck pathologies of hospitalized patients before and during the COVID-19 pandemic

DISEASES	Pre-COVID-19 Time^**a**^	COVID-19 Time^**b**^	Percentage decrease (%)
**Adenoid hypertrophy/adenotonsillar hypertrophy**	108	4	−96.3%
**Airways obstruction (dyspnea)**	63	53	−15.9%
**Benign tumors of head and neck**	64	39	−39.1%
**Congenital cysts and fistulas of the neck**	4	0	−100%
**Diseases of the paranasal sinuses and tympanomastoid cavity requiring surgery**	116	32	−72.4%
**Dentofacial deformity**	66	31	−53.0%
**Epistaxis**	16	8	−50.0%
**Pharyngotonsillitis/ peritonsillar abscess**	23	11	− 52.2%
**Malignant neoplasms of the head and neck**	58	56	−3.7%
**Maxillofacial trauma**	71	38	−46.5%
**Miscellanea**	373	170	−54.4%
**Nasal bone fractures**	36	12	− 66.7%
**Nasal deformities/septal deviation**	33	0	−100%
**Otitis media**	27	7	−74.1%
**Otitis media with effusion** **(ear tube insertion)**	17	0	−100%
**Vertigo**	45	8	−82.2%

^a^Pre-COVID-19 Time: March 1st to November 30th, 2019

^b^COVID-19 Time: March 1st to November 30th, 2020

Additionally, the Head and Neck team designated two maxillofacial surgeons and two otolaryngologists to be in charge of consultations for the hospitalized COVID-19 patients, as well as for the tracheostomies in COVID-19 patients in the intensive care unit (ICU). Tracheostomies have been performed by the Head and Neck team in 23 COVID-19 patients thus far. However, according to the call to action from the European Laryngological Societies [23], the recommendation of our institution was to delay tracheostomy until the patient no longer requires ventilation in the prone position. Therefore, the main indication for tracheostomy was limited to COVID-19 patients with prolonged endotracheal intubation lasting more than 14–21 days. Patients discharged from the ICU are then scheduled for a strict ORL follow-up in cooperation with the Laryngologist in our department to monitor the potential occurrence of laryngotracheal complications (i.e., stenosis, granulomas, etc.).

Lastly, the pre-COVID-19 number of hospital beds was 34 and has been reduced by 50% during the pandemic. Therefore, the current number of beds in the Head and Neck Department is 16 plus one bed available for urgencies/emergencies. No more than one patient in a double room and two patients in a quadruple room are being hospitalized in phase 1 and 2 in order to guarantee adequate distance between individuals. One empty bed is left between one patient and another whenever possible or at least 2 m of space between beds. As an additional safe distancing measurement, patients’ beds are placed diagonally [24].

### Organization of the outpatient clinic

During the Italian lock-down, which limited people’s movement and coincided with the “phase 1 protocol”, the role of the telephone triage was fundamental. Dedicated personnel were in charge of ascertaining the absence of signs/symptoms of COVID-19 and establishing the urgency/priority of the visit. With the “phase 2 protocol”, the activities of the outpatient clinic gradually returned back to the ordinary routine and patients on the waiting list were recalled. Twenty outpatients per day (Monday-Friday) were usually scheduled before the COVID-19 pandemic. The number of visits was reduced to 6 during the “phase 1 protocol” and almost returned to normal during the “phase 2 protocol” with 18 outpatients visited per day. Plexiglass protective barriers have been installed in order to prevent the spread of respiratory droplets and protect patients and personnel. According to the type and complexity of the visit, a waiting time ranging from 20 to 40 min is guaranteed between one patient and another in order to sanitize and aerate the room.

In accordance with the guidelines of the SIOeChCF [25], the health care workers admitted to the outpatient clinic should be limited to the essential (a nurse, an attending and a resident). The temperature check is done immediately upon arrival. Only the patient is admitted to the visit; visitors are not allowed, except for children and patients with disabilities. Physician and patient should maintain one-meter-distance in between each other during the interview. Empty seats should be left in the waiting room in order to ensure social distancing.

Both health care professionals and the patient should wash and / or sanitize their hands before the visit starts. Personnel should wear a disposable water-repellent gown, face shield, FFP2 or preferably FFP3 mask, cap and non-sterile gloves. During the visit it is important that the patient wears a surgical mask that should only be removed when necessary.

### Organization of the operating setting

According to the guidelines of the SIOeChCF [21], patients have to undergo a swab test for the detection of SARS-CoV-2 infection within 48 h before surgery. When, for any reason, it is not possible to execute a swab test, the patient should be considered positive for COVID-19. When patients test negative, high-level PPE are not required, while in cases of positive or suspected patients, the personnel should be limited to the essential and wear double gowns, double gloves, shoe cover, double cap, FFP2 or possibly FFP3 mask covered by a surgical mask and face shield.

Negative pressures in the operating rooms are fundamental in limiting the diffusion of pathogens, including SARS-CoV-2.

The use of debriders, drills, powered instruments and electrocoagulation should be limited or avoided. When this is not possible, the use of high-level PPE together with COVID-19 patient screening is strongly recommended [26]. The same PPE are warranted for the team executing tracheostomies in COVID-19 patients. Further precautions are trying not to puncture the cuff of the tracheostomy tube and preferring cuffed non-fenestrated tubes.

Moreover, especially during these critical times, it is important for head and neck surgeons to work in cooperation with the ICU and check the availability of beds before performing any complex surgical procedures (i.e., TORS, free flap surgery, laryngectomy).

### Toward the health system of the future: implementing telehealth systems in the COVID-19 era

In the last few years telehealth systems have become increasingly important and more and more sophisticated; likely, telehealth consultations are going to become ordinary in medicine in the near future and patients will be increasingly followed virtually. This implementation will help reduce the costs and inconvenience of travelling and clinics will be able to receive external technical support very quickly. Additionally, physicians and therapists will be able to provide care remotely and guarantee access to high-level services even to underserved and rural areas [27].

In the era of “social distancing”, the potentialities of eHealth and telemedicine appear even more promising and our institution is also taking advantage of these systems. In fact, during the “phase 1 protocol”, the CI team in the Head and Neck Department performed the first “remote fitting” [28] of a CI system. The implanted patient was a woman living outside of the Puglia region who underwent CI surgery in March 2020, prior to the Italian lockdown. Activation, mapping and measurements were performed by the CI team during a telehealth session preventing the patient from having to travel a distance during the critical phase of lockdown. In-person visits gradually started to be scheduled again with the activation of “phase 2 protocol”, but the” remote fitting” system was successfully tested and is ready for an eventual return to the “phase 1 protocol”.

Additionally, telehealth consultations played a central role in the management of head and neck cancer patients. The Department supported a campaign promoted by the Italian Association of Head and Neck Oncology (AIOCC) [29] to raise awareness for head and neck cancer tumors. Patients were invited to fill out an online questionnaire and subjects at risk were automatically scheduled for a free telehealth consultation and/or an in-person visit.

## Discussion and conclusions

The fear of this “new COVID-19 era” should not determine a “new cancer era”. Head and neck surgeons know how crucial early diagnosis is in oncological patients, as it implies higher probability of treatment success. Therefore, when counseling head and neck cancer patients, the take home message of this first year of the COVID-19 pandemic should be “the sooner, the better”. From our experience, one of the greatest difficulties was the cancer screening, as patients tend to seek medical attention at a more advanced disease stage or when they have already developed life-threatening complications. In our opinion, the fear of acquiring a nosocomial COVID-19 infection together with the inappropriate organization of many hospitals played a central role in this delay. Conversely, the oncological patients already scheduled for surgical operations experienced a shorter waiting time than the pre-COVID-19 era due to the cancellation/reduction of elective procedures [30].

One year since the first outbreak in Wuhan, China, we now have a clear understanding that the COVID-19 pandemic is going to be a long-term situation with lasting effects on public health and the entire society [31]. Therefore, an efficient health care system has to adopt a double strategy in this historical moment: always being ready for a surge of COVID-19 cases, while not forgetting non-COVID-19 patients, among whom, head and neck cancer patients represent a priority. Distinct from the articles [32] and guidelines [33] published during the first months of the pandemic, this review presenting the “COVID-19 organization protocol” of the research hospital “Casa Sollievo della Sofferenza” offers a different and enduring perspective accompanied by a new awareness of the disease and a gradual return to elective procedures. The COVID-19 pandemic has undoubtedly only accelerated the process toward the health care system of the future, where telemedicine [34], artificial intelligence [35] and mobile applications [36] are going to have more and more use.

## Data Availability

Supplementary data and material are available upon request to the corresponding author.

## Abbreviations

COVID-19: Coronavirus Disease 2019
SARS-CoV-2: Severe acute respiratory syndrome coronavirus 2
SIOeChCF: Italian Society of Otolaryngology-Head and Neck Surgery
ORL: Otolaryngology
ENT: Ear, Nose and Throat
URTI: Upper respiratory tract infections
ORL-HNS: Otolaryngology-head and neck surgery
AGPs: Aerosol-generating procedures
YO-IFOS: Young Otolaryngologists–International Federation of Otolaryngologic Societies
ISS: Higher Institute of Health
CI: Cochlear implantation/implant
PPE: Personal protective equipment
AJCC: American Joint Committee on Cancer
OPHL: Open partial horizontal laryngectomy
CT: Computed tomography
BAL: Bronchoalveolar lavage
FESS: Functional endoscopic sinus surgery
DCR: Dacryocystorhinostomy
TORS: Transoral Robotic Surgery
OSAS: Obstructive sleep apnea syndrome
AHI: Apnea hypopnea index
ICU: Intensive care unit
AIOCC: Italian Association of Head and Neck Oncology
